# The Influence of Type 2 Diabetes and Glucose-Lowering Therapies on Cancer Risk in the Taiwanese

**DOI:** 10.1155/2012/413782

**Published:** 2012-06-05

**Authors:** Ming-Chia Hsieh, Tzu-Chi Lee, Shu-Min Cheng, Shih-Te Tu, Ming-Hong Yen, Chin-Hsiao Tseng

**Affiliations:** ^1^Division of Endocrinology and Metabolism, Department of Internal Medicine, Changhua Christian Hospital, Changhua, Taiwan; ^2^Graduate Institute of Integrated Medicine, China Medical University, Taiwan; ^3^Department of Public Health, Kaohsiung Medical University, Kaohsiung, Taiwan; ^4^School of Pharmacy, College of Pharmacy, Kaohsiung Medical University, Kaohsiung, Taiwan; ^5^Department of Internal Medicine, National Taiwan University College of Medicine, No. 7 Chung-Shan South Road, Taipei 100, Taiwan

## Abstract

*Objective*. To investigate the association between type 2 diabetes, glucose-lowering therapies (monotherapy with either metformin, sulphonylurea or insulin) and cancer risk in Taiwan. *Methods*. Using Taiwan's National Health Research Institutes database of 1,000,000 random subjects from 2000–2008, we found 61777 patients with type 2 diabetes (age ≥20 years) and 677378 enrollees with no record of diabetes. *Results*. After adjusting for age and sex, we found patients with diabetes to have significantly higher risk of all cancers (OR: 1.176; 95% CI: 1.149–1.204, *P* < 0.001). Diabetic patients treated with insulin or sulfonylureas had significantly higher risk of all cancers, compared to those treated with metformin (OR: 1.583; 95% CI: 1.389–1.805, *P* < 0.001 and OR: 1.784; 95% CI: 1.406–2.262, *P* < 0.001). Metformin treatment was associated with a decreased risk of colon and liver cancer compared to sulphonylureas or insulin treatment. Sulfonylureas treatment was associated with an increased risk of breast and lung cancer compared to metformin therapy. *Conclusions*. Taiwanese with type 2 diabetes are at a high risk of breast, prostate, colon, lung, liver and pancreatic cancer. Those treated with insulin or sulfonylureas monotherapy are more likely to develop colon and liver cancer than those treated with metformin.

## 1. Introduction

Cancer has become the leading cause of death in Taiwan since 1982 [1]. Tseng [2] reported cancer to be the second leading cause of mortality in patients with type 2 diabetes in Taiwan. Patients with type 2 diabetes are known to be at increased risk of cancer and cancer mortality [3–9], especially hepatic [3] pancreatic [6], colon [8], bladder [9–11], and breast cancer [5, 12]. The relationship between type 2 diabetes and cancer is complex, possibly involving insulin resistance, hyperinsulinemia, and elevated levels of insulin-like growth factor-1 (IGF-1) in tumor cell growth [13, 14].

Glucose-lowering therapy may also play a role in the relationship between type 2 diabetes and cancer. Metformin treatment might reduce the risk of tumor development [15–20], whereas insulin and sulphonylureas might increase the risk [21, 22]. This study used Taiwan's National Health Insurance claims database to investigate the relationship between type 2 diabetes, glucose-lowering therapy with either metformin, sulphonylureas, or insulin alone, and cancer in the Taiwanese.

## 2. Methods

### 2.1. Data Sources

Taiwan's National Health Insurance (NHI) medical claims database, including ambulatory care, hospital inpatient care, dental services, and prescription drugs, was provided by Taiwan's National Health Research Institutes (NHRI). NHI coverage rate was 96.16% of the whole population in 2000 and rose to 99% by the end of 2004. The data set used for this study was a randomly sampled cohort of 1 million individuals enrolled in the NHI system from 2000 to 2008. It included information on registration entries, ambulatory care claims, inpatient care claims, and prescription. Patient identification numbers were scrambled for protection of confidentiality, and hence no ethics board approval was needed.

 NHI diagnosis coding follows the International Classification of Diseases, Ninth Revision (ICD-9), Clinical Modification diagnostic criteria. Records of claims for diabetes care were collected for patients with diabetes-related diagnosis with ICD-9 code 250 (excluding type 1 diabetes with ICD-9 code 250.1). An individual was classified as a diabetic patient if she or he had an initial diabetes-related diagnosis at any time in 2000 and then had at least one service claim from either ambulatory or inpatient care within the subsequent twelve months. Focusing on newly diagnosed cancer cases, we excluded patients diagnosed for any type of cancer (ICD-9: 140–209, 230–239) before the first year of the study period (2000-2001). The end of study period for each enrollee, both diabetic and nondiabetic, was, if any, first episode of primary or secondary diagnosis of any types of cancer from 2002 to 2008.

 The previous studies revealed that metformin treatment [15–20] might reduce the risk of tumor development and insulin and sulphonylurea [21, 22] might increase it. Our study wanted to clarify the influence of these antidiabetic agents (metformin, insulin, and sulphonylurea) on cancer risk in Taiwanese. To investigate the association between antidiabetic agents (monotherapy with either metformin, sulphonylurea, or insulin) and incident cancer, we included only type 2 diabetic patients receiving monotherapy with either metformin, sulphonylurea, or insulin. The kind of insulin included intermediate/long acting human insulin (HI), insulin glargine, insulin detemir, fast acting HI and insulin analogues, and premix HI and insulin analogues. The kind of sulphonylurea included glibenclamide, gliclazide, glipizide, and glimepiride. These patients had to have received continuous drug coverage for at least one year during study period and have no prior diagnosis of cancer. We excluded patients who were diagnosed as having cancer before the time they were prescribed antidiabetic drugs during the study period. The male patients were excluded from our analysis of breast cancer, and the female patients from our analysis of prostate cancer.

### 2.2. Statistical Analysis

 The risk of type 2 diabetes on cancers was tested by logistic regression models with age and sex adjustment. To determine the independent effects of antidiabetic drugs on the risk of any types of cancers, we used logistic regression models with age and sex adjustment. Breast cancer and prostate cancer were only adjusted for age but not sex. All statistical operations were performed using SAS version 9.2. A *P*-value of less than 0.05 was considered significant.

## 3. Results

In total, 61777 patients with type 2 diabetes (mean age 61.44 ± 13.23 years; 51.1% male) were followed up from 2000 to 2008. Patients with type 2 diabetes were found to be at a significantly higher risk of all cancers (odds ratio (OR): 1.176; 95%, confidence interval (CI): 1.149–1.204, *P* < 0.001) after adjusting for sex and age while compared to nondiabetic subjects (Table 1). Female and elderly subjects were at a significantly higher risk of all cancers than their male and younger counterparts (OR: 1.293, 95% CI: 1.273–1.313, *P* < 0.001 and OR: 1.040, 95% CI: 1.039–1.040, *P* < 0.001, resp.).

As can be seen in Table 2, a summary of incident cases of different types of cancer in patients with and without diabetes, patients with type 2 diabetes were at significantly higher risk of breast cancer, prostate cancer, colon cancer, lung cancer, liver cancer, and pancreatic cancer after adjusting for sex and age as compared to nondiabetic subjects.

A total of 10189 patients with type 2 diabetes (mean age 61.18 ± 14.03 years; 52.2% male) were identified as receiving monotherapy of insulin, sulfonylureas, or metformin. Patients receiving insulin or sulfonylurea had a higher risk of all cancers, compared to those receiving metformin (OR: 1.583, 95% CI: 1.389–1.805, *P* < 0.001 and OR: 1.784, 95% CI: 1.406–2.262, *P* < 0.001, resp.), after adjusting for sex and age (Table 3). Female patients with type 2 diabetes were at a significantly lower risk of all cancers than the male patients with type 2 diabetes (OR: 0.777, 95% CI: 0.692–0.873, *P* < 0.001). Elderly patients with type 2 diabetes were at a significantly higher risk of all cancers (OR: 1.037, 95% CI: 1.033–1.042, *P* < 0.001) as compared to younger patients with type 2 diabetes.

Table 4 shows the adjusted odds ratios for specific cancers associated with antidiabetic drugs. Patients receiving insulin or sulphonylureas had a higher risk of colorectal and liver cancers compared to those receiving metformin after adjusting sex and age. Sulphonylureas were additionally associated with an increased risk of breast and lung cancer. We found no relationship between glucose-lowering therapy and prostate, esophageal, gastric, or pancreatic cancer.

## 4. Discussion

 Our study demonstrates that Taiwanese with type 2 diabetes are at a high risk of cancer, especially breast, prostate, colon, lung, liver, and pancreatic cancer compared to nondiabetic subjects (Tables 1 and 2). Among diabetic patients, those receiving insulin or sulphonylurea monotherapy are at a higher risk of cancer compared to those receiving metformin (Table 3). Patients treated with metformin are at a lower risk of colorectal and liver cancers, compared to those receiving either insulin or sulphonylurea (Table 4), and at a lower risk of breast and lung cancer (Table 4), compared to those receiving sulphonylureas.

 Type 2 diabetes has already been linked to an increased risk of cancer [3–12]. One meta-analysis [5] found the relative risk (RR) of breast cancer to be 1.20 for women with diabetes compared to women without diabetes. Three meta-analyses found RR of colon, pancreatic, and hepatocellular cancer in diabetic patients to be 1.30 [8], 1.82 [6], and 1.84 [3], compared to nondiabetic subjects. However, the incidence rates of type 2 diabetes and cancer vary widely across populations. The current study found people with diabetes in Taiwan to be at a high risk of all cancers (odds-ratio (OR): 1.176; 95%, confidence interval (CI): 1.149–1.204, *P* < 0.001), especially breast, colon, liver, lung, prostate, and pancreatic cancers (Table 2). Recently, Lee et al. [23] also reported patients with diabetes to be at a high risk of liver, colon, lung, and prostate cancer. Our study found that patients with type 2 diabetes were not at high risk of gastric cancer (Table 2). Recently, Tseng [24] reported that diabetic Taiwanese have a higher risk of gastric cancer mortality. It should be pointed out that overall incidence of gastric cancer and mortality from the disease are two different entities and probably linked to different factors.

 Previous studies [25–28] have shown that cancer incidence is much higher in males than females at nearly all ages. Our study revealed that female Taiwanese have higher incidence of cancers as compared to male subjects in the general population (Table 1). However, we found that female diabetic patients have lower incidence of cancers as compared to male diabetic patients (Table 3). The sex disparities in cancer incidence might be due to illness behavior, health care access and utilization and ethnic difference [29, 30]. Future epidemiologic studies should be encouraged to design, analyze, and report sex-specific associations to aid the understanding of sex differences in cancer incidence in Taiwanese.

The association between diabetes and cancer may be mediated by metabolic syndrome and obesity through hyperinsulinemia and insulin resistance. Insulin is a growth hormone and is known to have atherogenic and mitogenic properties [31–33]. One observational study showing a relationship between level of circulating insulin and cancer has suggested that cancer growth may be influenced by the insulin-IGF-1 signaling axis [34].

This study found male Taiwanese with diabetes to be at a higher risk of prostate cancer than nondiabetic subjects (OR: 1.137, 95% CI: 1.036–1.249, *P* = 0.007, Table 2). This is inconsistent with Tseng's finding of a positive association between diabetes and prostate cancer in Taiwan, an association that became more remarkable in the younger patients [35]. However, previous studies of populations with European ancestries [36, 37] have reported men with diabetes to have a 20% lower risk of developing prostate cancer than men without diabetes. Two large-scale population-based cohort studies in Japan [38, 39] found no relationship between diabetes and prostate cancer, and one recent study [40] has suggested that diabetes is a protective factor for prostate cancer across populations, including Japanese Americans. The possible reasons for these inconsistent results may be ethnic and environment factors, screening frequency of prostate cancer, and the use of prostate specific antigen.

Our study also found that diabetic Taiwanese were at a high risk of lung cancer (OR: 1.296, 95% CI: 1.214–1.384, *P* < 0.001, Table 2). Coughlin et al. [4] reported diabetic men as well as women to be at a higher risk of lung cancer in the US. Jee et al. [41], studying UK population, also found slightly higher but insignificant risk of lung cancer for men and significantly higher risk for women, after adjusting for age, age squared, smoking, and drinking. Some cohort studies, however, have reported a negative association between diabetes and lung cancer [42–44]. Given these inconsistent findings, further prospective studies are needed to confirm the relationship between type 2 diabetes and specific cancers in different ethnic populations.

Our study found that diabetic patients treated with sulphonylurea or insulin monotherapy were at a significantly higher risk of cancers, compared to those treated with metformin. Likewise, Currie et al. [45] have also found diabetic patients on insulin or insulin secretagogues to be more likely to develop solid cancers than those on metformin. Bowker et al. [46] reported that patients with type 2 diabetes treated with sulphonylureas and insulin are at significantly increased risk of cancer-related mortality than those treated with metformin. Our finding that not only treatment with insulin but also treatment with sulphonylurea increased the risk seems to exclude an adverse property of the insulin formulation itself. Our study revealed that there was no significant association between antidiabetic agents (monotherapy with either metformin, sulphonylurea, or insulin) and prostate cancer in Taiwanese with type 2 diabetes. Tseng [47] also reported that insulin use is not significantly predictive for prostate cancer mortality in diabetic Taiwanese. Recently, Lai et al. [48] also reported that the use of metformin would decrease the risk of lung cancer in Taiwanese with diabetes. Taken together, these studies strongly suggest that glucose-lowering agents may play a role in the relationship between type 2 diabetes and some cancers. How they do this remains unclear. Metformin decreases insulin resistance and may thus possibly reduce the risk of cancer. It might also directly act on AMP-activated protein kinase (AMPK) signaling pathway [17].

Compared to those treated with sulphonylureas or insulin, diabetic patients treated with metformin had a significantly lower risk of developing colorectal and liver cancers (Table 4). Currie et al. [45] also found an association between metformin use and a lower risk of colon cancer. We further revealed that metformin was associated with a lower risk of breast and lung cancer, compared to sulphonylureas (Table 4). These findings suggest that metformin may have anticancer effects, sufficient to justify its use as a first-line treatment for diabetes and its potential use outside the context of diabetes.

This study has several limitations. First, it is subject to many limitations inherent to all observational studies. We lacked information on potentially important clinical covariates, such as smoking status, weight or body mass index, glycemic control, and alcohol consumption. Second, patients might be prescribed different treatment regimens for health-related reasons. Third, there were relatively small numbers of some specific cancers in patients with different glucose-lowering therapy, thereby limiting the power of our analysis.

In conclusion, type 2 diabetes is a risk factor for cancer in the Taiwanese. Metformin use was associated with a lower risk of cancer of the colon and liver, two common cancers in Taiwan. Further prospective studies are needed to confirm this relationship and study the possible mechanisms between cancers and antidiabetic drugs in Taiwan.

## Figures and Tables

**Table 1:** Adjusted odds ratios and 95% confidence intervals for all cancers associated with type 2 diabetes, sex, and age.

Covariate	Adjusted odds ratio	95% CI	*P* value
Type 2 diabetes (versus nondiabetic subjects)	1.176	1.149–1.204	<0.001
Sex (female versus male)	1.293	1.273–1.313	<0.001
Age (every 1-yr increment)	1.040	1.039–1.040	<0.001

**Table 2:** Adjusted odds ratios and 95% confidence intervals for specific types of cancer associated with type 2 diabetes.

Site-specific cancer	Patients with type 2 diabetes		Subjects without diabetes		Adjusted odds ratio		*P* value
	(*n* = 61777)		(*n* = 677378)		(95% CI)		
	Cases	%	Cases	%			
Breast	665	2.20	4042	1.16	1.111	(1.018–1.212)	0.018
Prostate	587	1.86	2223	0.68	1.137	(1.036–1.249)	0.007
Colon	1739	1.98	7219	1.07	1.206	(1.142–1.274)	<0.001
Lung	1226	2.81	4281	0.63	1.296	(1.214–1.384)	<0.001
Liver	1528	2.47	5558	0.82	1.582	(1.491–1.680)	<0.001
Stomach	523	0.85	2700	0.40	0.920	(0.836–1.012)	0.088
Pancreas	286	0.46	731	0.11	2.038	(1.768–2.349)	<0.001

Subjects without diabetes as reference and adjustment for sex and age.

**Table 3:** Adjusted odds ratios and 95% confidence intervals for all cancers associated with antidiabetic drugs, sex, and age in type 2 diabetic patients treated with metformin, sulfonylurea, or insulin monotherapy.

Covariate	Adjusted odds ratio	95% CI	*P* value
Antidiabetic drugs			
Sulfonylurea versus metformin	1.784	1.406–2.262	<0.001
Insulin versus metformin	1.583	1.389–1.805	<0.001
Sex (female versus male)	0.777	0.692–0.873	<0.001
Age (every 1-yr increment)	1.037	1.033–1.042	<0.001

**Table 4:** Adjusted odds ratios and 95% confidence intervals for specific cancers associated with antidiabetic drugs.

Site-specific cancer	Metformin		Sulfonylurea		Insulin		Age-sex-adjusted odds ratio	Age-sex-adjusted odds ratio
	(*n* = 3963)		(*n* = 6072)		(*n* = 751)		(95% CI)	(95% CI)
	Cases/*n*	%	Cases/*n*	%	Cases/*n*	%	Insulin versus metformin	Sulfonylureas versus metformin
Breast*	19/2048	0.93	48/2804	1.71	5/338	1.48	1.630 (0.604–4.396)	1.765 (1.030–3.024)
Prostate*	27/1915	1.41	52/3268	1.59	5/413	1.21	0.893 (0.338–2.359)	1.034 (0.644–1.659)
Colon and rectum	46/3963	1.16	145/6072	2.39	18/751	2.40	2.135 (1.226–3.717)	1.847 (1.320–2.585)
Lung	45/3963	1.14	122/6072	2.01	9/751	1.20	1.058 (0.513–2.183)	1.570 (1.110–2.220)
Liver	58/3963	1.46	143/6072	2.36	19/751	2.53	1.818 (1.075–3.077)	1.504 (1.104–2.049)
Stomach	20/3963	0.50	54/6072	0.89	7/751	0.93	1.855 (0.779–4.419)	1.547 (0.923–2.594)
Pancreas	8/3963	0.20	21/6072	0.35	1/751	0.13	0.693 (0.087–5.545)	1.594 (0.705–3.619)

*Breast cancer and prostate cancer were only adjusted for age but not sex.
